# Supplementary material for: Thrombosis and Hyperinflammation in COVID-19 Acute Phase Are Related to Anti-Phosphatidylserine and Anti-Phosphatidylinositol Antibody Positivity
Source: Biomedicines. 2023 Aug 18;11(8):2301. doi: 10.3390/biomedicines11082301 (PMC10452204; doi:10.3390/biomedicines11082301)
Supplement: Supplementary file 1 [file biomedicines-11-02301-s001.zip › biomedicines-2548750-supplementary.pdf]

Supplementary Table S1. Laboratory data at hospital admission and 48 h of hospitalization of APLA positive and APLA negative patients.

	Reference values	APLA positive (N=30)†	APLA negative (N=62)†	*p*-Value‡
Haematocrit (%)	36.0-45.0			
Admission		41.0 (5.1)	41.5 (4.7)	0.75
48h		39.0 (4.5)	39.6 (4.1)	0.36
Haemoglobin (g/dl)	12.0-15.0			
Admission		13.9 (1.8)	13.8(1.9)	0.97
48h		13.3 (1.6)	13.2 (1.4)	0.55
MCV (fl)	80.0-98.0			
Admission		89.3 (6.9)	88.6 (4.9)	0.29
48h		89.2 (6.3)	87.8 (4.7)	0.26
Leukocytes (x10^9/l)	4.0-11.0			
Admission		6.2 (2.6)	6.3 (4.0)	0.88
48h		6.8 (3.8)	6.0 (3.4)	0.43
PMN (x10^9/l)	2.0-7.0			
Admission		4.5 (2.0)	4.7 (4.2)	0.95
48h		5.5 (3.2)	4.3 (2.6)	0.25
Lymphocytes (x10^9/l)	1.2-3.5			
Admission		0.9 (0.3)	0.9 (0.4)	0.89
48h		0.7 (0.4)	0.9 (0.6)	0.09
Monocytes (x10^9/l)	0.1-1.0			
Admission		0.4 (0.4)	0.5 (0.4)	0.62
48h		0.4 (0.3)	0.5 (0.3)	0.94
Platelets (x10^9/l)	150-400			
Admission		170.0 (100.5)	213.0 (102.3)	0.45
48h		189.0 (133.0)	234.0 (165.0)	0.32
PT (INR,ratio)	0.7-1.3			
Admission		1.14 (0.13)	1.12 (0.15)	0.53
48h		1.12 (0.18)	1.12 (0.15)	0.49
aPTT (seconds)	21-35			
Admission		29.0 (4.0)	28.0 (4.0)	0.07
48h		29.0 (5.0)	28.0 (5.8)	0.42
D-dimer (ng/ml)	<240			
Admission		399.0 (676.3)	298.0 (392.3)	0.17
48h		421.0 (520.0)	292.5 (428.5)	0.14
Creatinine (mg/dL)	0.51-0.91			
Admission		0.92 (0.29)	0.93 (0.34)	0.76
GFR ml/min/1.73m^2	>90			
Admission		78.12 (23.05)	83.16 (26.19)	0.56
LDH (IU/l)	120-246			
Admission		650 (265)	558 (291)	0.38
CRP (mg/l)	<10			
Admission		107.4 (85.2)	74.9 (130.4)	0.37
48h		89.4 (76.7)	90.2 (87.1)	0.88
Ferritin (ng/ml)	25-250			
Admission		620.0 (1331.0)	472.0 (496.0)	0.16
48h		525.0 (1279.8)	517.0 (611.0)	0.40
Procalcitonin (ng/ml)	<0.1			
Admission		0.1 (0.1)	0.1 (0.1)	0.56
48h		0.2 (0.1)	0.1 (0.1)	0.11
IL-6 (pg/ml)	<4.3			
Admission		55.3 (31.9)	36.0 (46.7)	0.18

48h		37.8 (61.4)	27.2 (52.7)	0.29

†Median (IQR); aPTT, activated partial thromboplastin time; CRP, C-reactive protein; GFR, glomerular filtration rate; IL-6, interleukin-6; LDH, lactate dehydrogenase; MCV, mean corpuscular volume; PMN, polymorphonuclear cells; PT, prothrombin time. ‡Mann-Whitney U test.
